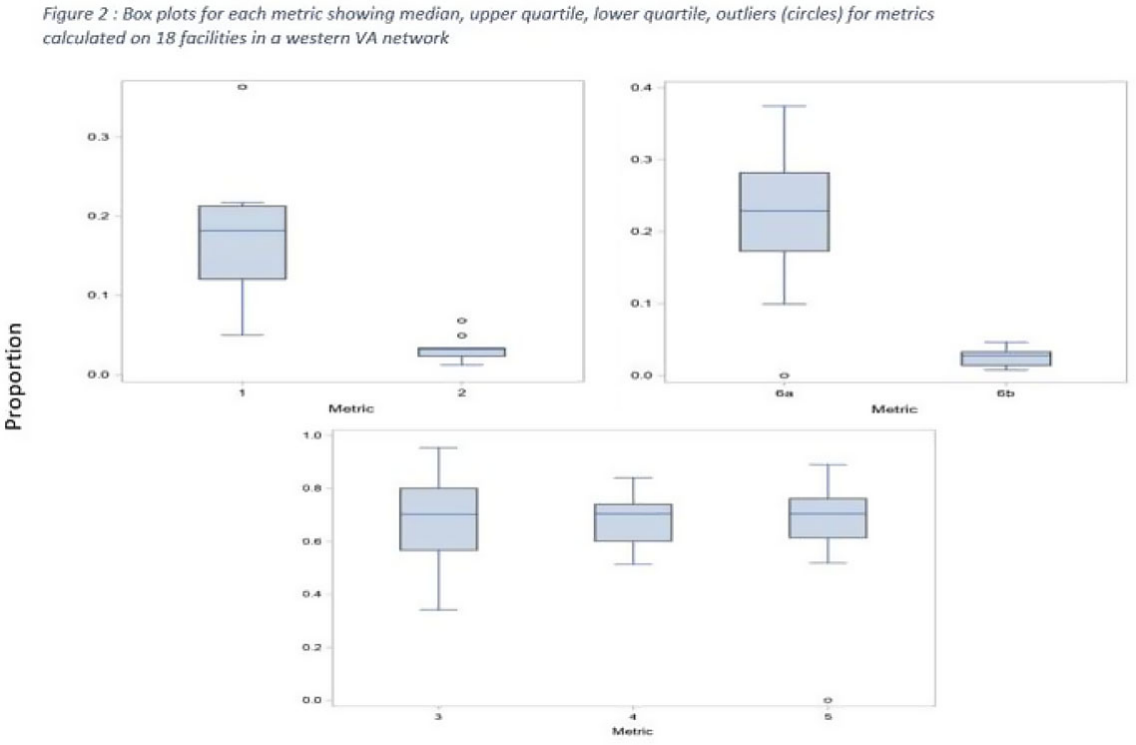# Tier-based antimicrobial stewardship metrics for genitourinary-related antibiotic use in Veterans’ Affairs outpatient settings

**DOI:** 10.1017/ash.2022.64

**Published:** 2022-05-16

**Authors:** Matthew Samore, Matthew Goetz, McKenna Nevers, Jacob Crook, Suzette Rovelsky, Ben Brintz, Kelly Echevarria, Melinda Neuhauser, Sharon Tsay, Lauri Hicks, Karl Madaras-Kelly

## Abstract

**Background:** Tracking antibiotic use is a core element of antimicrobial stewardship. We developed a set of metrics based on electronic health record data to support an outpatient stewardship initiative to improve management of urinary tract infections (UTIs) in Veterans’ Affairs (VA) emergency departments (EDs) and primary care clinics. Because UTI diagnostic codes only capture a portion of genitourinary (GU)-related antibiotic use, a tier-based approach was used to evaluate practices. **Methods:** Metrics were developed to target practices related to antibiotic prescribing and diagnostic testing (Table 1). GU conditions were divided into 3 categories: tier 1, conditions for which antibiotics are usually or always indicated; tier 2, conditions for which antibiotics are sometimes indicated; and tier 3, conditions for which antibiotics are rarely or never indicated (eg, benign prostatic hypertrophy with symptoms). Patients with visits related to urological procedures, nontarget providers, and concomitant non-GU infections were excluded. Descriptive analyses included calculation of the correlation matrix for the 7 metrics and the construction of box plots to display interfacility variability. **Results:** Metrics were calculated quarterly for 18 VA medical centers, including affiliated clinics, in a western VA network, from July 2018 to June 2020 (Table 1). Tier 3 GU conditions accounted for 1,276 of 11,840 (11%) of GU-related antibiotic use. Metrics 1 and 6b were strongly correlated with each other and were also positively correlated with metrics 2 and 5 (coefficients > 0.5) (Fig. 1). Substantial interfacility variation was observed (Fig. 2). **Conclusions:** Stewardship metrics for suspected or documented UTIs can identify opportunities for practice improvement. Broadly capturing GU conditions in addition to UTIs may enhance utility for performance feedback. Antibiotic prescribing for tier 3 GU conditions is analogous to unnecessary antibiotic use for acute, uncomplicated bronchitis and upper respiratory tract infections.

**Funding:** None

**Disclosures:** None

**Table:**
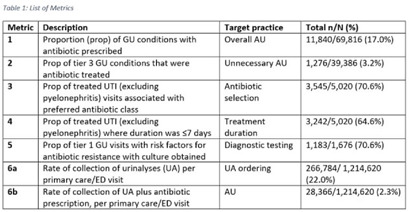

**Figure:**
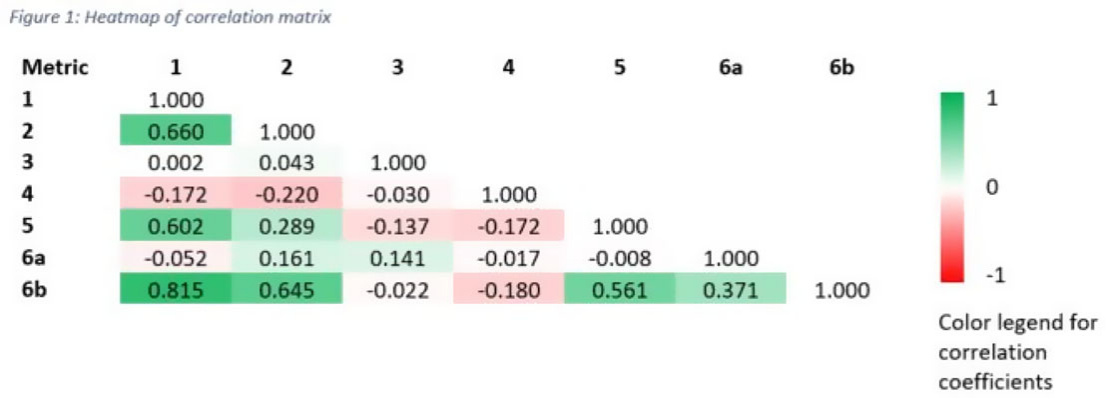

**Figure:**